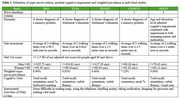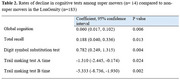# Risks of cognitive impairment and accelerated cognitive decline in super movers

**DOI:** 10.1002/alz70860_107185

**Published:** 2025-12-23

**Authors:** Oshadi M Jayakody, Ying Jin, Cuiling Wang, Helena M Blumen, Joe Verghese

**Affiliations:** ^1^ Albert Einstein College of Medicine, Bronx, NY, USA; ^2^ Department of Epidemiology and Population Health, Department of Neurology, Albert Einstein School of Medicine, New York, USA, Bronx, NY, USA; ^3^ Stony Brook Medicine, Stony Brook, NY, USA; ^4^ Stony Brook University, Stony Brook, NY, USA

## Abstract

**Background:**

Slow gait is a robust functional marker for dementia that identifies individuals’ risk for cognitive impairment up to a decade before a clinical onset. But, what about the other end of the spectrum? This abstract presents the results on whether “super movers” (a novel phenotype of individuals aged ≥80 years without dementia and with gait speeds ≥1.5 SD of sex‐adjusted cutoffs) have lower risks of cognitive impairment and/or slower cognitive decline.

**Methods:**

Data from five Health and Retirement Study International Network of Studies (HRS‐INS) were used to assess the risk of cognitive impairment in super‐versus non‐super movers. Cognitive impairment was defined as >1.5 SD below age‐adjusted cutoffs on at least one out of three tests, combined with impaired Instrumental Activities of Daily Living (Table 1). Age‐ and sex‐adjusted hazard ratios (HR) from cox proportional hazards models were pooled in a meta‐analysis to determine the overall risk for incident cognitive impairment. Data from the LonGenity study (a prospective longitudinal study in the Bronx) were used to examine the rates of decline in cognitive tests in super‐ versus non‐super movers using linear mixed effect models adjusted for age, sex, education and parental longevity,.

**Results:**

In individual HRS‐INS, super movers showed lower risks of cognitive impairment in the ELSA (English Longitudinal Ageing Study; HR 0.31, 95% CI 0.16‐0.60)) and SHARE studies (the Survey of Health, Ageing and Retirement in Europe HR 0.49, 95% CI 0.26‐0.89)). In the meta‐analysis, among 3,999 participants selected from 15,911=aged ≥ 65 (*n* = 358 super movers; baseline age 83.6‐84.4 years) followed for 3.8‐6.1 years, super movers had significantly lower risk of cognitive impairment (HR 0.49, 95% CI 0.28‐0.71) (Figure 1). In the LonGenity study, (*n* = 197, baseline age 84.6 years (SD 3.3)) super movers showed a slower decline in global cognition, total recall, digit symbol substitution test and Trial Making Tests A and B (Table 2).

**Conclusions:**

Super movers—a novel aging phenotype—show lower risks of cognitive impairment and slower cognitive decline, in memory and non‐memory related tests. Investigating the behavioral and biological factors contributing to this phenotype may reveal protective mechanisms against cognitive decline and dementia risk.